# Supplementary material for: Factors Affecting Incurred Pesticide Extraction in Cereals
Source: Molecules. 2023 Jul 31;28(15):5774. doi: 10.3390/molecules28155774 (PMC10420941; doi:10.3390/molecules28155774)
Supplement: Supplementary file 1 [file molecules-28-05774-s001.zip › molecules-2481495-supplementary.pdf]

# Factors Affecting Incurred Pesticide Extraction in Cereals

**Xiu Yuan [1], Chang Jo Kim [1], Won Tae Jeong [1], Kee Sung Kyung [2,*] and Hyun Ho Noh [1,*]**

[1] Residual Agrochemical Assessment Division, Department of Agro-Food Safety and Crop Protection,
National Institute of Agricultural Sciences, Wanju 55365, Republic of Korea; yx0219@korea.kr (X.Y.);
rlackdwh1@gmail.com (C.J.K.); shewaspretty@korea.kr (W.T.J.)

[2] Department of Environmental and Biological Chemistry, College of Agriculture, Life and Environment Science, Chungbuk National University, Cheongju 28644, Republic of Korea

* Correspondence: kskyung@cbnu.ac.kr (K.S.K.); noh1983@korea.kr (H.H.N.); Tel.: +82-63-238-3225 (H.H.N.); +82-43-261-2562 (K.S.K.)

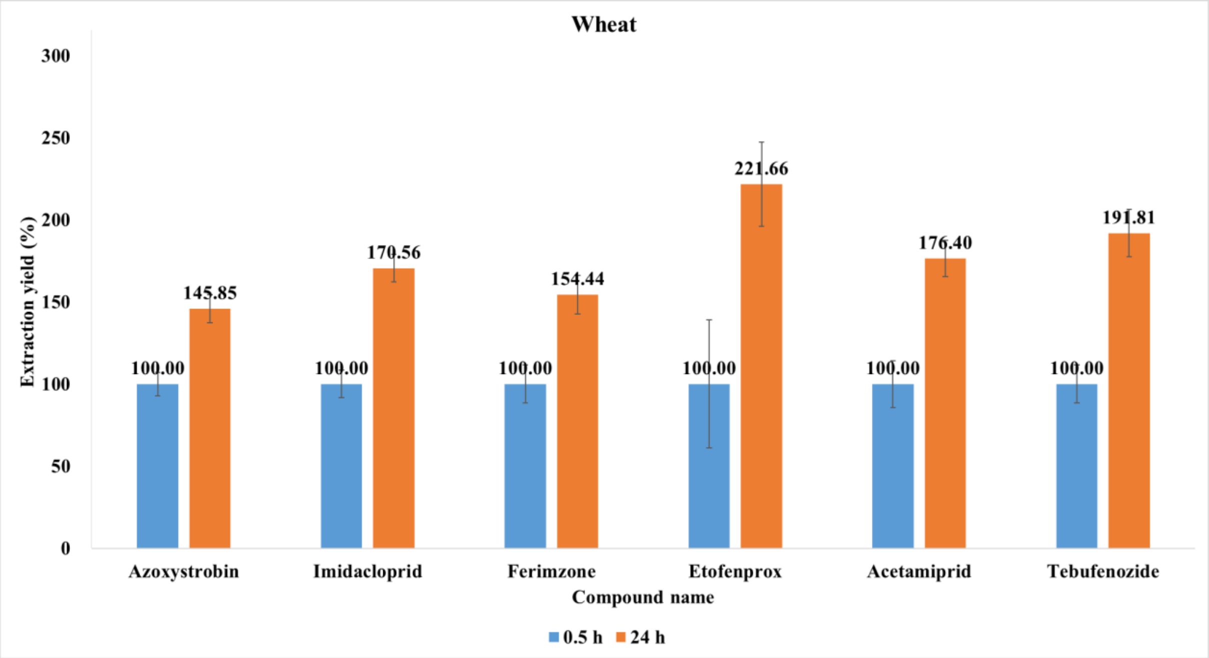


Figure S1. Immersion time optimization. The average extracted pesticide residues concentration at 0.5 h was set as the 100% extraction yield.

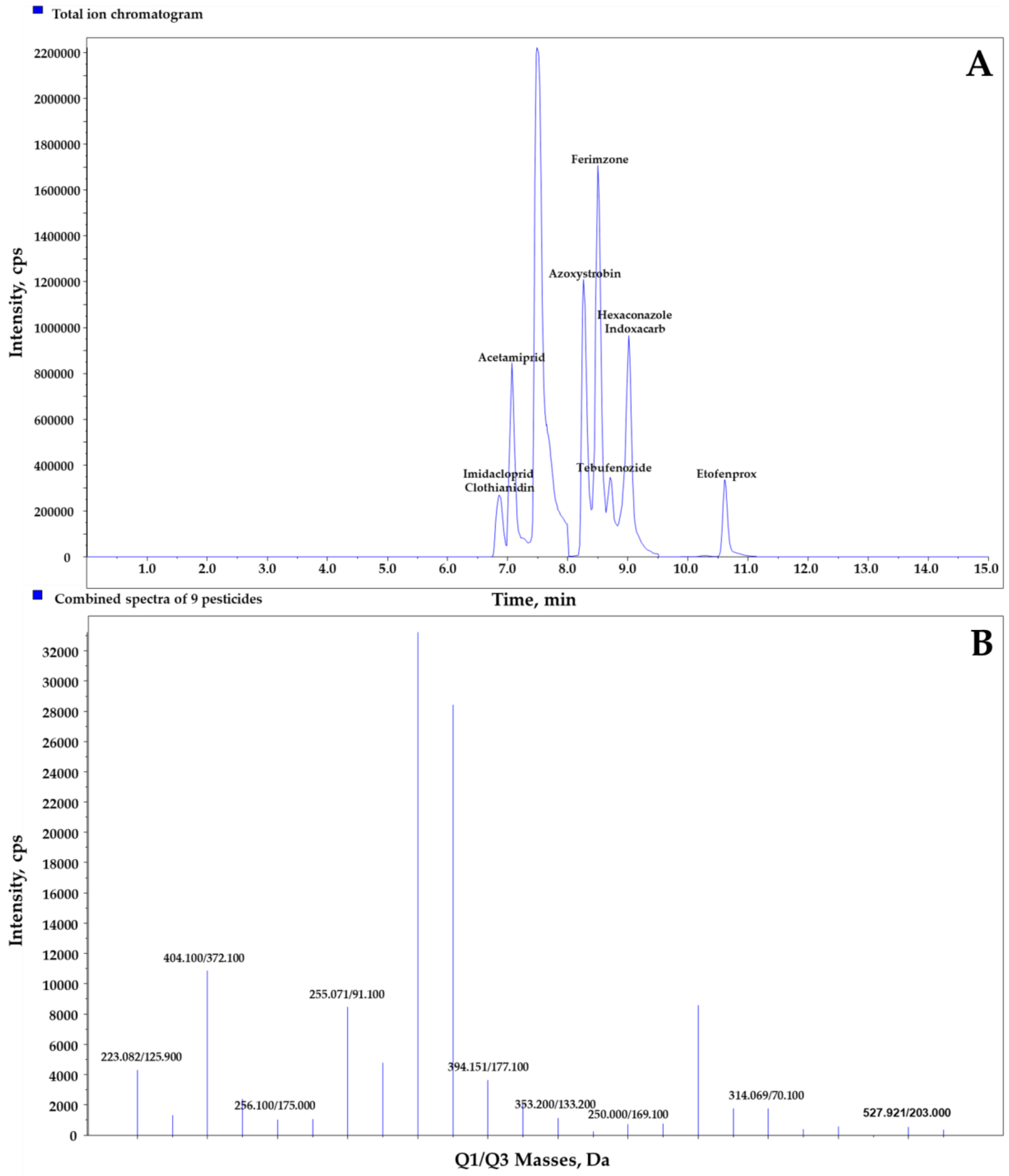


Figure S2. Total ion chromatogram (A) and combined spectra of 9 pesticides (B) at 50 ppb in rice.

**Table S1.** Method validation for rice, wheat, barley, and oat

| Matrix | Compound | $LOQ_{method}$ (ng/g) | $R^2$ | Linear range (μg/kg) | 10 ng/g | | 50 ng/g | | Matrix effect (%) |
|---|---|---|---|---|---|---|---|---|---|
| | | | | | Recovery (%) | RSD (%) | Recovery (%) | RSD (%) | |
| Rice | Acetamiprid | 10 | 0.997 | 1–50 | 97.31 | 0.41 | 115.93 | 0.53 | -17.4 |
| | Azoxystrobin | | 0.999 | | 114.24 | 0.75 | 117.88 | 1.08 | -20.4 |
| | Imidacloprid | | 0.998 | | 103.17 | 3.75 | 115.79 | 0.53 | -16.6 |
| | Ferimzone | | 0.993 | | 88.46 | 3.14 | 117.44 | 2.21 | -38.3 |
| | Etofenprox | | 0.994 | | 86.63 | 4.85 | 92.58 | 6.01 | -35.5 |
| | Tebufenozide | | 0.999 | | 94.86 | 12.87 | 105.31 | 5.22 | -20.5 |
| | Clothianidin | | 0.998 | | 103.58 | 1.05 | 117.14 | 0.20 | -26.1 |
| | Hexaconazole | | 0.998 | | 99.55 | 4.53 | 116.98 | 0.29 | -27.1 |
| | Indoxacarb | | 0.992 | | 90.33 | 3.01 | 107.96 | 0.81 | -17.4 |
| Wheat | Acetamiprid | 10 | 0.999 | 1–50 | 98.63 | 0.87 | 112.59 | 4.68 | -9.8 |
| | Azoxystrobin | | 0.998 | | 91.27 | 1.78 | 115.35 | 4.12 | -13.6 |
| | Imidacloprid | | 0.999 | | 111.46 | 1.86 | 111.57 | 4.36 | -8.0 |
| | Ferimzone | | 0.999 | | 81.28 | 3.75 | 117.66 | 3.22 | -30.4 |
| | Etofenprox | | 0.999 | | 105.86 | 1.81 | 104.50 | 3.91 | -25.2 |
| | Tebufenozide | | 0.997 | | 103.65 | 9.87 | 114.15 | 6.00 | -12.1 |
| | Clothianidin | | 0.999 | | 102.77 | 0.44 | 108.60 | 3.04 | -13.7 |
| | Hexaconazole | | 0.999 | | 95.27 | 11.20 | 106.96 | 5.21 | -10.9 |
| | Indoxacarb | | 0.999 | | 102.92 | 1.06 | 115.39 | 3.31 | -9.8 |
| Barley | Acetamiprid | 10 | 0.999 | 1–50 | 88.30 | 1.37 | 107.65 | 1.54 | -1.1 |
| | Azoxystrobin | | 0.997 | | 82.10 | 2.18 | 116.18 | 1.77 | -6.6 |
| | Imidacloprid | | 0.999 | | 92.11 | 4.32 | 105.45 | 3.44 | -1.4 |
| | Ferimzone | | 0.993 | | 96.84 | 1.56 | 116.10 | 2.09 | -31.2 |
| | Etofenprox | | 0.999 | | 116.82 | 2.12 | 104.54 | 4.61 | -25.0 |
| | Tebufenozide | | 0.997 | | 84.06 | 0.90 | 117.73 | 4.50 | -5.4 |
| | Clothianidin | | 0.999 | | 93.55 | 0.75 | 105.95 | 2.63 | -19.7 |
| | Hexaconazole | | 0.999 | | 96.08 | 2.88 | 101.45 | 1.45 | -7.1 |
| | Indoxacarb | | 0.999 | | 94.22 | 1.09 | 101.31 | 2.41 | -1.1 |
| Oat | Acetamiprid | 10 | 0.999 | 1–50 | 97.10 | 0.86 | 106.90 | 1.11 | -7.2 |
| | Azoxystrobin | | 0.998 | | 89.61 | 2.01 | 112.64 | 1.21 | -6.8 |
| | Imidacloprid | | 0.999 | | 110.34 | 3.54 | 103.37 | 1.69 | -0.8 |
| | Ferimzone | | 0.993 | | 101.09 | 0.41 | 114.27 | 1.06 | -51.4 |
| | Etofenprox | | 0.998 | | 72.01 | 1.17 | 83.64 | 5.59 | -30.2 |
| | Tebufenozide | | 0.999 | | 104.02 | 1.67 | 97.63 | 4.49 | -9.6 |
| | Clothianidin | | 0.999 | | 101.64 | 2.83 | 100.54 | 1.66 | -14.7 |
| | Hexaconazole | | 0.999 | | 106.53 | 3.52 | 94.27 | 2.39 | -11.0 |
| | Indoxacarb | | 0.999 | | 110.39 | 1.29 | 111.82 | 1.00 | -7.2 |

RSD: Relative Standard Deviation

**Table S2.** MRM conditions

| Compound | Q1 Mass (Da) | Q3 Mass (Da) | Time (min) | DP (V) | EP (V) | CE (V) | CXP (V) |
|---|---|---|---|---|---|---|---|
| Acetamiprid | 223.082 | 125.900<br>90.100 | 7.00 | 96 | 10 | 29<br>43 | 10<br>8 |
| Azoxystrobin | 404.100 | 372.100<br>344.100 | 8.25 | 74 | 10 | 19<br>27 | 4<br>4 |
| Imidacloprid | 256.100 | 175.000<br>209.000 | 6.80 | 89 | 10 | 25<br>23 | 4<br>4 |
| Ferimzone | 255.071 | 91.100<br>65.100 | 8.50 | 121 | 10 | 45<br>73 | 8<br>6 |
| Etofenprox | 394.151 | 177.100<br>107.000 | 10.64 | 61 | 10 | 21<br>61 | 18<br>12 |
| Tebufenozide | 353.200 | 133.200<br>296.900 | 8.70 | 81 | 10 | 23<br>15 | 4<br>4 |
| Clothianidin | 250.000 | 169.100<br>132.000 | 6.80 | 94<br>71 | 10 | 21<br>35 | 4<br>4 |
| Hexaconazole | 314.069 | 70.100<br>159.000 | 8.90 | 106 | 10 | 25<br>43 | 6<br>12 |
| Indoxacarb | 527.920 | 203.000<br>149.900 | 8.86 | 166 | 10 | 57<br>33 | 22<br>18 |

DP: Declustering Potential

EP: Entrance Potential

CE: Collision Energy

CXP: Cell Exit Potential

**Table S3.** Average extracted pesticide residue concentrations by particle size

| Crop | Pesticide | Extracted pesticide residue by cereal particle size (mg/kg, n = 3) | | | | |
|---|---|---|---|---|---|---|
| | | <10-mesh | 10–20-mesh | 20–40-mesh | 40–60-mesh | > 60-mesh |
| Rice | Acetamiprid | 14.0 e | 29.6 d | 50.6 c | 89.9 b | 131.8 a |
| | Azoxystrobin | 14.1 d | 16.6 d | 45.6 c | 133.3 b | 246.9 a |
| | Imidacloprid | 21.4 e | 44.6 d | 73.4 c | 125.6 b | 183.5 a |
| | Ferimzone | 18.3 d | 28.0 d | 74.9 c | 197.2 b | 326.0 a |
| | Etofenprox | 5.9 c | 5.2 c | 8.0 c | 22.1 b | 42.8 a |
| | Tebufenozide | 5.6 c | 6.2 c | 18.6 c | 61.8 b | 138.8 a |
| | Clothianidin | 1.6 e | 2.3 d | 3.6 c | 5.1 b | 7.1 a |
| | Hexaconazole | 20.1 d | 29.1 d | 71.3 c | 183.9 b | 328.4 a |
| | Indoxacarb | 10.5 d | 11.1 d | 38.7 c | 153.9 b | 318.6 a |
| Wheat | Acetamiprid | 38.1 d | 67.9 c | 87.8 a | 86.8 a | 77.4 b |
| | Azoxystrobin | 24.1 d | 40.3 c | 70.6 b | 83.0 a | 74.5 b |
| | Imidacloprid | 44.1 d | 77.3 c | 100.6 a | 99.0 a | 89.8 b |
| | Ferimzone | 47.2 c | 88.1 b | 106.5 a | 109.8 a | 86.4 b |
| | Etofenprox | 19.6 d | 22.3 d | 42.5.c | 61.6a | 56.7 b |
| | Tebufenozide | 35.2 e | 45.8 d | 69.6 c | 101.6 a | 81.3 b |
| | Clothianidin | 7.4 c | 12.9 b | 14.7 b | 18.2 a | 14.2 b |
| | Hexaconazole | 31.4 d | 56.6 c | 72.8 ab | 76.3 a | 61.5 bc |
| | Indoxacarb | 47.8 e | 60.7 d | 92.9 c | 131.4 a | 117.2 b |
| Barley | Acetamiprid | 41.4 e | 56.6 d | 99.6 c | 116.5 b | 116.5 a |
| | Azoxystrobin | 24.6 e | 32.6 d | 109.7 c | 175.3 b | 192.8 a |
| | Imidacloprid | 43.0 e | 56.0 d | 101.4 c | 117.4 b | 118.7 a |
| | Ferimzone | 29.2 e | 43.8 d | 124.1 c | 162.4 b | 167.4 a |
| | Etofenprox | 31.0 e | 34.2 d | 104.2 c | 206.1 b | 232.3 a |
| | Tebufenozide | 45.8 e | 48.8 d | 154.0 c | 289.7 b | 309.2 a |
| | Clothianidin | 5.4 e | 7.4 d | 13.1 c | 14.3 b | 14.4 a |
| | Hexaconazole | 20.8 e | 26.7 d | 73.9 c | 97.0 b | 104.6 a |
| | Indoxacarb | 43.2 e | 49.9 d | 181.3 c | 346.3 b | 394.1 a |
| Oat | Acetamiprid | 22.9 c | 59.6 a | 62.7 a | 58.3 ab | 51.4 b |
| | Azoxystrobin | 18.4 d | 39.9 c | 49.7 b | 59.6 a | 57.9 a |
| | Imidacloprid | 28.6 c | 69.5 a | 73.5 a | 69.4 a | 59.6 b |
| | Ferimzone | 19.0 c | 68.6 ab | 79.9 a | 76.1 ab | 66.5 b |
| | Etofenprox | 16.6 c | 20.9 bc | 26.4 b | 36.9 a | 37.4 a |
| | Tebufenozide | 17.6 d | 24.3 cd | 27.6 c | 44.1 b | 53.2 a |
| | Clothianidin | 3.1 c | 7.5 a | 8.1 ab | 7.4 ab | 6.5 b |
| | Hexaconazole | 12.1 d | 37.8 c | 48.2 a | 46.6 ab | 41.3 bc |
| | Indoxacarb | 38.3 c | 46.9 c | 66.1 b | 89.7 a | 93.8 a |

Notes: Same lowercase letters across a row indicate no significant differences among residue concentrations of same pesticide ($p < 0.05$). Extraction efficiency: a > b > c > d > e
